# Universal, Rapid, and Cleavable Labeling of Antibodies by Fluorophores and DNA Oligonucleotides for Multiplex Immunostaining and Spatial Proteomics Through MIST Linker

**DOI:** 10.3390/bios16070385

**Published:** 2026-07-15

**Authors:** Arafat Meah, Shuo Yin, Saimoen Strrrz Anderson, Ming Lin, Shuo Liang, Meghana Davuluri, Yi-Xian Qin, Sandeep K. Mallipattu, Jun Wang

**Affiliations:** 1 Multiplex Biotechnology Laboratory, Department of Biomedical Engineering, State University of New York at Stony Brook, Stony Brook, NY 11794, USA; arafat.meah@stonybrook.edu (A.M.); yinshuo0921@gmail.com (S.Y.); saimoen.anderson@stonybrook.edu (S.S.A.); shuo.liang@stonybrook.edu (S.L.); 2 Department of Computer Science, State University of New York at Stony Brook, Stony Brook, NY 11794, USA; ming.lin@stonybrook.edu; 3 Department of Biomedical Engineering, State University of New York at Stony Brook, Stony Brook, NY 11794, USA; meghana.davuluri@stonybrook.edu (M.D.); yi-xian.qin@stonybrook.edu (Y.-X.Q.); 4 Division of Nephrology and Hypertension, Department of Medicine, Stony Brook School of Medicine, Stony Brook, NY 11794, USA; sandeep.mallipattu@stonybrookmedicine.edu; 5 Renal Section, Northport VA Medical Center, Northport, NY 11768, USA; 6 MIST Bioscience, New York, NY 11794, USA

**Keywords:** MIST Linker, spatial proteomics, single cell analysis, nanobody labeling, photocleavable linker, antibody labeling

## Abstract

Direct antibody labeling is essential for immunoassays, multiplexed imaging, and biosensing; however, current methods are often time-consuming, restricted by antibody source, risk compromising protein performance, or vary with multiple steps. We introduce multiplex in situ tagging (MIST) Linker, a rapid and Fc-site-specific labeling tool that conjugates fluorophores or DNA oligonucleotides to antibodies from diverse commercial sources in as fast as 10 min using minimal starting material. MIST Linker achieves >90% cleavage upon UV exposure, facilitating rapid cyclic imaging on a single specimen. Validated across multiple species and sources, the platform outperforms conventional two-step immunofluorescence and immunohistochemistry in various tissues and cell lines. By enabling the rapid, cost-effective customization of antibody panels, MIST Linker significantly lowers the barrier to accessing antibody–DNA conjugates for spatial biology. When integrated with the spatial MIST platform and MIST-Explorer, it enables high-plex, single-cell spatial proteomics at high signal-to-noise ratios in human clinical biopsies, mouse specimens and cell lines. This toolkit provides an efficient, accessible solution for high-resolution spatial mapping, allowing for the in-depth analysis of cell subpopulations, biomarker distributions, and signaling events in complex biological specimens. Thus, MIST Linker offers a versatile, accessible, and scalable solution for antibody-labeling-based research and clinical diagnosis.

## 1. Introduction

Single-cell technologies have transformed biomedical research by enabling molecular profiling across heterogeneous cell populations. Most platforms focus on RNA and DNA readouts, where sequencing effectively serves as a high-throughput array to quantify genome-wide gene expression [1,2]. However, proteins, the primary effectors of cellular function, remain harder to measure at comparable scale particularly in intact tissues. Because protein abundance and spatial context more directly reflect cell state, they are central to diagnosis, cell type classification, and therapeutic targeting [3,4]. Protein-level profiling is especially important in disease and pathway studies, where mRNA and protein correlations can be weak, particularly for low-abundance signaling proteins [5,6,7]. These gaps have driven rapid development of high-plex proteomic technologies aimed at scaling protein measurements while preserving cellular and tissue context.

Antibody-based protein detection and quantification remain the gold standard and a routine tool for most biomedical studies, despite significant advancements in mass spectrometry (MS) in recent years. Biomarker assays on tissue or cell samples typically require multiple steps, including incubation with a primary antibody, a secondary antibody labeled with biotin, a fluorophore, or HRP, and potentially further steps to visualize the signal. Thus, a typical immunostaining procedure requires at least three hours after sample fixation and permeabilization. This required time is proportionally longer when multiple rounds of staining are needed in multiplexed assays. Advanced multiplex methods typically rely on primary antibodies conjugated with fluorophores, metal isotopes, or oligonucleotides. Techniques like cyclic immunofluorescence, IBEX, and CODEX perform repeated rounds of staining and imaging to expand multiplexing, enabling panels of more than 60 markers in a single sample within about a week [5,6,7,8]. Imaging mass cytometry and multiplexed ion beam imaging use metal-isotope-labeled antibodies to quantify roughly 40 markers in tissue sections [9,10,11], while the Orion platform captures up to 18 fluorescence channels in a single imaging pass [12]. The highest multiplexity, achieved by single-cell multiplex in situ tagging (MIST), has reached the quantitation of 404 different proteins in single cells [13]. Concurrently, MS-based methods, which do not require antibodies for protein recognition, have advanced significantly. Emerging spatial MS techniques, such as deep visual proteomics (DVP), in situ imaging proteomics via expansion (iPEX), and parallel-flow projection and transfer learning across omics data (PLATO), can detect over 1000 protein types, sometimes even at subcellular resolution [14,15,16,17]. However, they suffer from a major drawback: the majority of well-recognized biomarker proteins remain undetectable. MS essentially analyzes digested peptides that may not uniquely map to a particular protein [16]. Due to limited sensitivity, detecting only one or two distinct peptides cannot robustly reveal a protein identity; consequently, only a few hundred structural proteins are typically determined with high confidence. Furthermore, these methods are inherently destructive, and the tissue cannot be reused.

Direct labeling of primary antibodies can significantly shorten immunohistochemistry (IHC) and immunocytochemistry (IC) procedures, yet it remains a persistent challenge in the field. Most chemical labeling approaches randomly target free amine groups for conjugation; however, antibody antigen-binding sites are often naturally rich in these reactive groups [18,19]. The conjugated moieties, such as oligonucleotides or small proteins, can also introduce steric hindrance, thereby compromising or completely blocking the antibody’s binding affinity. Thus, to preserve functionality, modifications should ideally be restricted to the Fc region of the IgG molecule, spatially isolating the conjugate from the Fab binding sites. This site-directed approach is particularly critical when tagging antibodies with DNA oligonucleotides, which can introduce substantial structural bulk relative to the antibody itself [20]. Antibody–DNA conjugates are widely utilized in immunopolymerase chain reaction (immuno-PCR), proximity ligation assays (PLAs), multiplexed assay development, cellular targeting technologies, CITE-seq experiments, in situ sequencing (ISS), and epigenetic assays [20,21,22]. Currently, the majority of commercial conjugation kits rely on stochastic chemical reactions and require relatively large starting quantities (e.g., ~50 µg) of highly purified antibody [23]. These purified, carrier-free formulations are expensive and frequently unavailable from major vendors. Consequently, failed conjugations incur substantial financial and temporal losses in multiplex assay development, a challenge our laboratory has acutely experienced while attempting to conjugate over two thousand antibodies over the past decade.

Antibody–DNA conjugates (ADCs) are also critical to the workflow of single-cell MIST, which currently achieves the highest multiplexity among single-cell proteomic assays. The core components of MIST technology include a highly compact DNA barcode array and custom-designed, ultraviolet (UV)-cleavable single-stranded DNA (ssDNA)–antibody conjugates [24,25]. This platform effectively translates protein detection into DNA detection, leveraging DNA-based techniques for highly multiplexed analysis. Herein, we report a novel ADC approach, termed MIST Linker, that enables the one-step, rapid conjugation of DNA oligonucleotides to antibodies from diverse sources. Remarkably, as little as 1 μg of starting antibody is sufficient for successful labeling of DNA oligonucleotides and subsequent immunostaining of a standard tissue section. We have validated this approach across various cell lines and tissue types, demonstrating its superior performance compared to conventional two-step immunofluorescence (IF) and immunohistochemistry (IHC). Furthermore, the MIST Linker is rapidly cleaved by UV irradiation within 10 min at an efficiency exceeding 90%, thereby facilitating cyclic, reiterative IF on the same tissue specimen over multiple rounds. When combined with MIST arrays, we demonstrate that this updated spatial MIST platform achieves high sensitivity and spatial resolution comparable to our established spatial MIST assays utilizing conventional chemically conjugated ADCs. We further demonstrate the utility of spatial MIST in conjunction with MIST-Explorer for the analysis of a mouse cell line, human colon cancer tissues, and kidney biopsy samples. Given these compelling features, the spatial MIST platform built upon the MIST Linker approach is poised to find broad utility in fundamental biomedical research, clinical diagnostics and prognostics, and drug discovery and evaluation.

## 2. Materials and Methods

### 2.1. Preparation of DNA Conjugated Microbeads

Amine-functionalized polystyrene microbeads (2 μm; Life Sciences Technologies, Carlsbad, CA, USA) were conjugated with two single-stranded DNA (ssDNA) sequences at equal amounts per bead. Oligonucleotides were custom designed and purchased from Integrated DNA Technologies, with each sequence assigned to a specific protein target. DNA conjugation was performed using DBCO azide click chemistry as previously described [26]. All oligonucleotide sequences are listed in Table S1.

Briefly, 100 μL of amine-bearing microbeads were incubated with 10 mM bis(sulfosuccinimidyl)suberate (BS3; Thermo Fisher Scientific, Waltham, MA, USA) for 20 min, followed by centrifugation and washing with PBST (PBS containing 0.05% Tween 20). Beads were then treated with 0.05% poly L lysine (PLL; Ted Pella Inc., Redding, CA, USA) for 2 h on an orbital shaker, washed with PBST, and incubated with 5 mM Azido PEG4 NHS (Vector Laboratories) for 4 h under continuous agitation. In parallel, each ssDNA sequence (150 μM) was reacted with DBCO NHS (Click Chemistry Tools, Newark, CA, USA) at a final concentration of 5 mM in pH 8.5 buffer for 4 h at room temperature. After reaction completion, microbeads were washed with PBST and modified oligonucleotides were purified using a 7 kDa MWCO Zeba spin column (Thermo Fisher Scientific, Waltham, MA, USA). Purified DNA was then incubated overnight with the functionalized beads under continuous shaking. After conjugation, beads were washed with Milli-Q water and resuspended to the original concentration.

For array fabrication, DNA-functionalized beads were mixed in equal proportions and assembled on glass slides as previously reported [13,24]. Arrays were examined by microscopy to confirm monolayer formation and uniform bead distribution (Figure S1).

### 2.2. Preparation of Antibody Assembly

Fc-specific MIST Linkers were prepared by click chemistry crosslinking. The resulting conjugate was purified by fast protein liquid chromatography (FPLC) and concentrated to 1 mg/mL using a 10 kDa MWCO centrifugal filter. For assembly of the final staining reagent, antibody (1 μL, 1 mg/mL) was incubated with 3 μL of MIST Linker conjugate for 10 min at room temperature. Next, 2 μL of 50 mM biotinylated oligonucleotide carrying a matched fluorophore label (Alexa Fluor 555 or Alexa Fluor 647) at the opposite end was added and incubated for an additional 0.5 h. After purification by a desalt column, the purified conjugates were pooled into an antibody cocktail and passed through a 100 kDa MWCO centrifugal filter. The final volume was adjusted to approximately 50 μL using blocking buffer containing 5% goat serum and fragmented salmon sperm DNA, and the resulting reagent was used for tissue staining and MIST microarray analysis.

Antibody conjugation using Lightning-Link^®^ Cy5 Antibody Labeling Kit (Abcam Cambridge, UK; catalog #703-0010) and oYo-Link^®^ Antibody Conjugation Reagents (AlphaThera, Philadelphia, PA, USA) was performed according to the manufacturers’ instructions.

For native polyacrylamide gel electrophoresis (Native PAGE), 1.25 μg of each sample (5 μL) was premixed with 5 μL of sample loading buffer (Invitrogen, Thermo Fisher Scientific, Waltham, MA, USA; catalog #LC2673) and loaded onto 3–8% Tris-acetate gels (Invitrogen, Thermo Fisher Scientific, Waltham, MA, USA; catalog #EA0375BOX). Two independent gels were analyzed. The first gel included the unbound antibody and the fully formed MIST Linker complex as controls. The second gel was used to evaluate MIST Linker complex formation after reaction times of 30 s, 1 min, 5 min, 10 min, and 20 min. The assembled complex was frozen to stop the reaction at each time point, then thawed before loading into the gel. Electrophoresis was performed using Tris-Glycine Native Running Buffer (Invitrogen, Thermo Fisher Scientific, Waltham, MA, USA; catalog #LC2672under native conditions at 80 V for 25 min, followed by 120 V for 110 min. The electrophoresis apparatus was maintained in an ice bath throughout the run to preserve native protein complexes and minimize heat generation. After electrophoresis, the gels were stained and destained with Coomassie G-250 (Invitrogen, Thermo Fisher Scientific, Waltham, MA, USA; catalog #LC6060) according to the manufacturer’s instructions.

### 2.3. Tissue Processing

FFPE tissue sections, including human kidney biopsy, human lung tumor biopsy, and wildtype mouse kidney samples, were processed using common dewaxing, antigen retrieval, and blocking workflow. Dewaxing and antigen retrieval were performed with an automated heat induced epitope retrieval (HIER) module and the manufacturer’s dedicated retrieval buffer (Epredia, Portsmouth, NH, USA), which standardized temperature, timing, and buffer composition across runs. The program began at 85 °C, increased gradually to 98 °C, included a 20 min incubation at 98 °C, and then cooled back to 85 °C. Following retrieval, sections were washed in PBS for 5 min and permeabilized in 0.3% Triton X-100 in PBS for 10 min. Tissue sections were then blocked for 1 h at room temperature in PBS containing 5% goat serum, 100 μg/mL fragmented salmon sperm DNA (Thermo Fisher Scientific, Waltham, MA, USA), 0.05% Tween 20, and a cocktail of unlabeled blocking oligonucleotides. The blocking oligonucleotides carried the same sequences as the antibody-associated oligonucleotides listed in Table S1, but lacked the biotin tag, and were included at a final concentration of 10 μM to reduce nonspecific DNA-mediated interactions.

### 2.4. MC3T3 and MDA-MB-231 Cell Culture and Processing

Glass coverslips were coated with poly L lysine for 2 h, washed, air dried, and placed individually into wells of a 12-well plate. MC3T3-E1 cells (ATCC CRL-2593) and MDA-MB-231 cells (ATCC HTB-26) were obtained from ATCC (Manassas, VA, USA). Cells were seeded onto the coverslips in complete growth medium containing antibiotics and cultured at 37 °C in a humidified 5% CO_2_ incubator for 3 days, until approximately 50% confluency was reached. Cells were then fixed with 4% formaldehyde for 10 min at room temperature, washed with PBS, permeabilized with 0.3% Triton X 100 in PBS for 10 min, and washed again with PBS. Cells were subsequently blocked for 1 h at room temperature using the same blocking buffer as used for tissue sections. The blocking buffer consisted of PBS containing 5% goat serum, 100 μg/mL fragmented salmon sperm DNA (Thermo Fisher Scientific, Waltham, MA, USA), 0.05% Tween 20, and a cocktail of unlabeled blocking oligonucleotides matching the antibody-associated oligonucleotide sequences listed in Table S1, but lacking the biotin tag, at a final concentration of 10 μM.

### 2.5. Staining, Clamping, and MIST Array Imaging of Tissue Sections and Cultured Cells

After blocking, tissue sections and cultured MC3T3 cells on coverslips were incubated for 3 h in the dark at room temperature with antibody–MIST Linker complexes at a final antibody concentration of approximately 0.02 mg/mL per antibody. Complete antibody and oligonucleotide information is provided in Tables S1 and S2. Antibody panels were prepared based on the sample type and experimental context. A full list of antibodies is shown in Table S2. Following incubation, samples were washed three times with PBST and stained with the nuclear dye POPO-1 (Invitrogen) at 1:200 dilution in PBST for 15 min. This step enabled nuclear labeling and facilitated transfer of spatial information onto the MIST array during clamping.

In parallel, MIST arrays were blocked with PBST for 10 min, washed three times with PBST, and incubated with 30% (*w*/*v*) 8 arm PEG OH (40 kDa, Creative PEGWorks, Chapel Hill, NC, USA) for 10 min to reduce nonspecific interactions. Tissue sections or coverslips containing cultured cells were then carefully aligned with the MIST arrays, secured using a magnetic clamp, and exposed to 365 nm UV light for 5 min (CS2010 UV Curing System; Thorlabs, Newton, NJ, USA). UV exposure facilitated cleavage and transfer of the oligonucleotide barcodes, as well as transfer of the nuclear dye from the sample to the array. After UV exposure, the arrays and samples were separated and washed thoroughly with PBST. Arrays were mounted and imaged directly after clamping using an Olympus VS200 slide scanner (Evident Scientific, Tokyo, Japan). Imaging was performed sequentially in brightfield for array registration, DAPI for POPO-1 nuclear signal, Cy3 for Alexa Fluor 555-labeled targets, and Cy5 for Alexa Fluor 647-labeled targets. Images were computationally aligned and analyzed using custom in-house software to localize and quantify protein signals.

### 2.6. MIST Array Decoding Process

MIST arrays were decoded either before or after protein detection, as previously described [13,24]. Briefly, arrays were treated with 1 M NaOH for 2 min to dissociate double-stranded DNA complexes, followed by five washes with PBST. Arrays were then hybridized for 1 h at room temperature with fluorophore-labeled complementary DNA (cDNA) probes at 200 nM in SSC buffer containing 40% formamide and 10% dextran sulfate. After three additional PBST washes, arrays were reimaged to complete the first decoding cycle. A second decoding cycle was then performed using a different set of fluorophore-labeled cDNA probes under the same conditions. Although all samples were decoded using two cycles, the decoding scheme was customized for each sample according to its antibody panel and the number of protein targets analyzed. Bead identities were assigned based on the panel-specific fluorescence sequence observed across the two decoding cycles. The predefined fluorescence codes corresponding to each protein marker are listed in Table S3.

### 2.7. MIST Array Registration and Image Analysis

Images acquired during protein detection and decoding were computationally aligned using a custom image processing pipeline developed in house [27]. Brightfield images from Cycle 2 and protein detection were registered to the Cycle 1 brightfield image as the reference. To improve alignment efficiency, images were cropped and rotated before registration. Because local distortions in the TIFF images limited the accuracy of single-transformation-based approaches, conventional feature-based methods such as SIFT and SURF were insufficient. Therefore, a pixel-level alignment strategy was used to achieve bead-level registration accuracy.

To streamline image alignment, cell segmentation, and visualization of protein expression, we developed a Python (v3.10.18 )-based program, MIST-Explorer. The program uses a tiling strategy to align large images and stitch them together, resulting in final registration accuracy within 1 pixel. Following registration, beads with detectable signals in the protein channel were identified, and their fluorescence transitions across the two decoding cycles were used to assign protein identity. Beads were then mapped to segmented cells, and their associated protein expression values were recorded. A threshold was applied in the protein channel to exclude background noise. When multiple beads corresponding to the same protein were detected within a single cell, the median bead intensity was used as the representative expression value. The aligned TIFF images were subsequently converted into CSV files containing single-cell spatial coordinates and protein expression values on a 16-bit scale.

Cell segmentation was performed on aligned images using StarDist (v2.9) with the 2D fluorescence model (2D_versatile_fluo) and default settings, including percentile normalization from the 1st to 99.8th percentile, a nucleus probability threshold of 0.5, and a nonmaximum suppression threshold of 0.4 [28]. These are available within MIST-Explorer. Segmentation outputs were manually assessed for each sample by overlaying predicted nuclear boundaries onto the DAPI channel. Poorly segmented cells and cells with extremely low total signal were excluded from downstream analysis. Cell centroid coordinates were recorded as Global X and Global Y for subsequent spatial analyses.

### 2.8. Single-Cell Data Preprocessing and Statistics

Single-cell data were filtered to remove background noise and imaging artifacts. Signal intensities were adjusted by subtracting the background level plus two standard deviations to estimate true protein expression values. Expression values were log2 transformed and z score normalized before clustering and other downstream analyses. Single-cell protein expression matrices were analyzed in R using Seurat (v4.0) which is also available on MIST-Explorer. UMAP analysis was performed using only the 10 phenotype-defining protein markers, such that each cell was represented by a 10-dimensional expression vector. Spatial coordinates (Global X and Global Y) were excluded from PCA and UMAP and used only to map cells back to their original tissue locations. Graph-based Louvain clustering was then performed at a resolution of 0.3 to balance cluster granularity with biological interpretability. UMAP dimensionality reduction was carried out using 15 neighbors, a minimum distance of 0.15, and the first 5 principal components, generating a two-dimensional representation of the cellular landscape. Cluster identities were assigned by examining the top differentially expressed proteins identified with FindAllMarkers and comparing them with canonical kidney cell markers.

### 2.9. Visualization of Cell–Cell Spatial Relationships

To characterize the spatial organization of protein-expressing cells, pairwise Euclidean distances were calculated between all segmented cells based on their spatial coordinates. These distances were used to generate a spatial proximity matrix representing the average separation between cells expressing each protein marker. For visualization, median pairwise distances for all protein combinations were first computed within each group and then averaged to establish the midpoint of the heatmap scale. A red-to-blue color gradient was applied, where values below the midpoint indicate closer spatial proximity (red) and values above indicate greater separation (blue). Spatial proximity analysis was performed across multiple protein panels, including 15 markers in kidney tissue (105 unique pairs) and 13 markers in the MC3T3 cell line (78 unique pairs). Approximately 12,000–18,000 single cells were analyzed per biopsy, with regions segmented into glomerular and interstitial compartments. For each cell, distances to all other cell types were computed, and mean proximity values were derived for every protein–protein pair.

### 2.10. Signal-to-Noise Quantification

Signal-to-noise (S/N) ratio was defined as:

$$S/N=\frac{\mathrm{Signal}-\mathrm{Background}}{\mathrm{Background}}$$

Signal intensity was determined as the mean fluorescence intensity within manually selected regions corresponding to positive staining, while background intensity was measured from manually selected regions lacking visible signal to account for nonspecific fluorescence and imaging noise. For each condition, S/N values were calculated across replicate measurements and averaged to obtain a mean S/N. Variability across replicates was quantified using the standard deviation (SD), which is shown as error bars in the corresponding plots. This approach enables consistent comparison of signal contrast across different staining conditions.

## 3. Results and Discussion

### 3.1. MIST Linker Design and Characterization by Immunostaining of Various Cell and Tissue Samples

**Figure 1:**
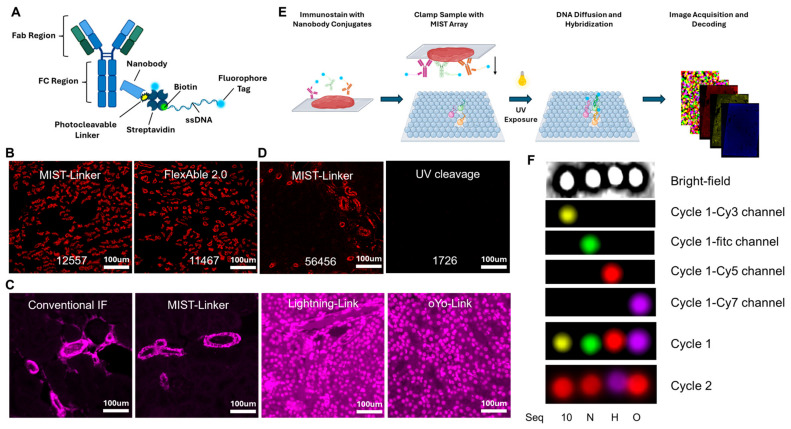
MIST Linker for rapid, cleavable labeling of antibodies and for integration in spatial MIST. (**A**) Schematic design of MIST Linker. (**B**) Comparison of MIST Linker and FlexAble 2.0 in immunostaining of Megalin on a human kidney sample. Average fluorescence intensities of stained features are labeled. (**C**) Immunostaining of mouse kidney tissue by anti-αSMA antibody through conventional two-step approach, and the same antibody labeled with one-step MIST Linker, Lighting-Link, and oYo-Link kit. (**D**) Comparison of fluorescence before and after UV cleavage of MIST Linker on a human kidney sample. Average fluorescence intensities of stained features are labeled. (**E**) Procedure of spatial MIST using MIST Linker for ADC conjugation. Image generated using BioRender. (**F**) Representative decoding colors on microbeads showing the unique combinations.

MIST Linker is composed of a nanobody and streptavidin connected by two photocleavable linkers, where streptavidin permits further convenient attachment of biotinylated molecules such as biotin-fluorophores and biotin-oligonucleotides. Selected nanobodies specifically bind to the Fc region of primary antibodies from mice and rabbits (Figure 1A). The 70 kDa MIST Linker appears to attach to both sides of the Fc region of an antibody as confirmed by protein gel electrophoresis. However, further conjugation of oligonucleotides may introduce steric hindrance. An improved 29 kDa MIST Linker from MIST Bioscience can access both sides. Because of the high affinity of the nanobody for the antibody Fc and the streptavidin for biotin, the MIST Linker enables the tagging of 1 μg of antibody with biotinylated molecules in as little as 10 min. Consistent with this, time course gel analysis showed formation of the antibody and MIST Linker complex as early as 30 s, with no detectable unassembled antibody or intermediate bands across time points from 30 s to 20 min (Figure S2). The performance of the MIST Linker is comparable to the commercial FlexAble 2.0 in the IF staining of Megalin, with fluorescence intensities of 12,557 versus 11,467 (Figure 1B). Additionally, the fluorescence intensity from MIST Linker labeling is comparable to conventional two-step IF staining on kidney tissue. High performance of MIST Linker is critical to the spatial MIST technology where the sensing mechanism is based on local capture of UV-cleaved oligonucleotides of conjugations from immunostained tissue, and the multiplexing is also relying on the orthogonality of those oligonucleotides (Figure 1E,F).

We further compared the performance of MIST Linker with current antibody–DNA conjugation (ADC) methods through immunostaining. The Lightning-Link conjugation kit is similar to the traditional chemical crosslinking approach that requires ideally pure antibody and oligonucleotides with no other reagents such as BSA or tris buffer carrying primary amines. It also requires at least 50 μg of antibody to start with to obtain purified conjugates with multiple steps of precipitation and resuspension in addition to temperature control, while the whole process spans over 4 h to overnight. The Fc-site-specific approaches represented by oYo-Link only target the Fc region of an antibody and require minimally 1 μg of input. This technique is based on affinity of protein A/G on the Fc region and modified protein sequence to incorporate photoreactive groups, and consequentially covalent bonds are formed toward antibodies. However, it might be more applicable to small-molecule conjugation, and it does not permit convenient custom synthesis of oligos in the laboratory. We used oYo-Link Azide to conjugate DBCO-modified oligonucleotides and then attach the conjugates to anti-mouse αSMA antibody with UV light exposure. Both Lightning-Link and oYo-Link show high background in immunostaining of mouse kidney samples even with 5% salmon sperm DNA blocking, which is likely due to lacking an efficient purification method to remove unbound oligonucleotides. On the contrary, with the same blocking reagents and experimental condition, we barely observed high nonspecific background signal on MIST Linker staining without purification. Further purification of conjugates by 40 KDa WMCO zeba columns effectively removed the excess of oligonucleotides, enabling multiplex immunostaining of conjugates.

**Figure 2:**
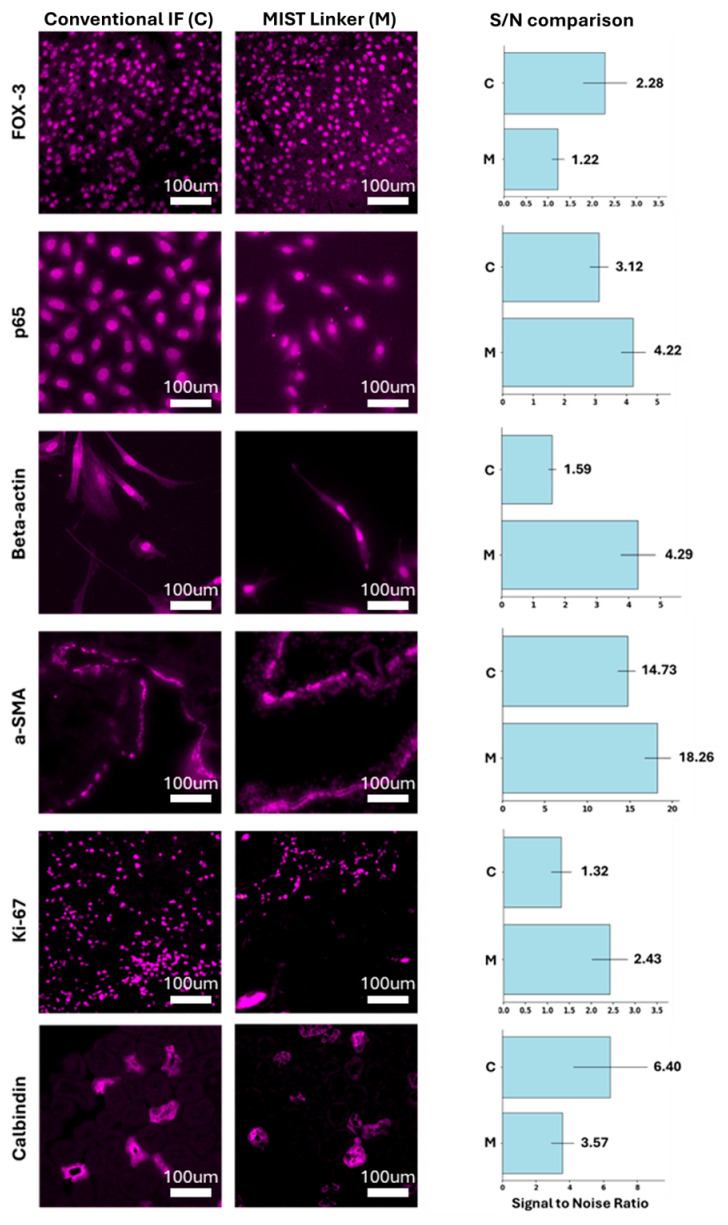
Comparison of conventional immunostaining (C) and MIST Linker staining (M) across multiple proteins and biological sample types. Images of immunostaining results of both methods are quantified, and signal-to-noise ratio for each of them has been quantified in the bar charts on the right. Error bars represent the standard deviation (SD) of means. From top to bottom on the left: FOX-3 on mouse brain, p65 on MDA-MB-231 cell line, β-actin on MC3Tc cell line, αSMA on human lung tumor biopsy, Ki-67 on human colon biopsy, and calbindin on mouse kidney.

The MIST Linker was validated through the immunostaining of various mouse and human tissue samples as well as cell lines (Figure 2). Antibodies including mouse IgG1, IgG2, and rabbit isotypes were selected to detect Fox3 in mouse brain, p65 in the MDA MB 231 cell line, beta actin in the MC3T3 cell line, αSMA in human lung tumor biopsies, Ki-67 in human colon biopsy, and calbindin in mice. Fluorescence intensities of the cells and stained features were quantified to compare conventional IF with MIST Linker methods. With the exception of Fox3 and calbindin, all tested proteins exhibited higher signal intensities with MIST Linker staining compared to conventional two-step IF, while about 1 μg of antibody input was used for both methods. The detection of nuclear proteins such as Ki67 and perinuclear proteins like p65 was not hindered by their cellular localization. MIST Linker staining remained consistent with conventional IF, confirming accurate epitope recognition. Notably, the MIST Linker required only half the time for immunostaining without compromising specificity or affinity. Negative control staining with the MIST Linker alone showed minimal nonspecific binding of the linker under these staining conditions (Figure S3). Although select targets showed reduced raw signal intensity with the MIST Linker (Figure S4), their spatial localization remained consistent with conventional IF, suggesting preserved target recognition while indicating that some antibody–linker combinations may require target-specific optimization to maximize signal. The high immunostaining intensity may be attributed to the multiple fluorophores carried by each MIST Linker molecule. Quantitative comparisons with commercial streptavidin, which carries three fluorophores, revealed that each MIST Linker molecule carries four or more fluorophores. Increasing the number of fluorophores does not necessarily increase fluorescence due to proximity-induced self-quenching, as observed in our experiments with Cy5 dyes. This quenching effect is alleviated by utilizing Alexa Fluor series dyes and MB dyes [29,30].

**Figure 3:**
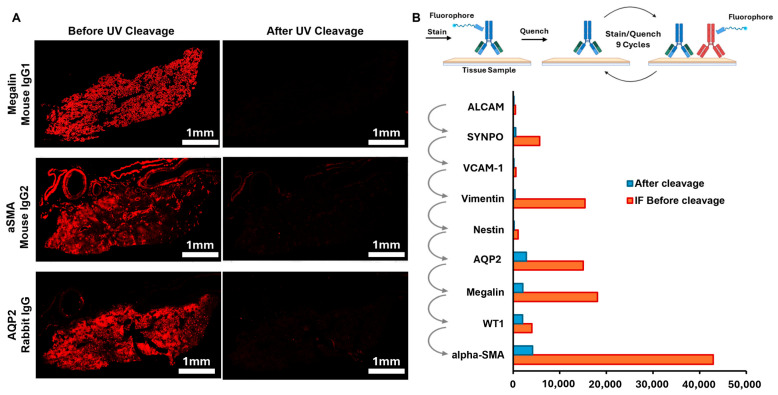
Cleavage of MIST Linker upon UV exposure and reiterative staining. (**A**) Representative images of immunostaining by MIST Linker-labeled Megalin, αSMA and AQP2 antibodies on human kidney samples before UV exposure (left panel) and after UV exposure (right panel). (**B**) Scheme of reiterative staining of MIST Linker-labeled antibodies on the same tissue for 9 cycles. The bar chart shows the fluorescence of immunostained features and the same features after quenching by UV exposure in each cycle starting from ALCAM.

MIST Linker demonstrates high potential for reiterative staining in multiplex assays. Human kidney tissue was stained with MIST Linker-labeled antibodies for one hour followed by UV cleavage before the next round of staining, which is similar to a cyclic IF procedure. Analysis of three kidney markers—megalin, alpha SMA, and AQP2—showed 96%, 93%, and 91% cleavage efficiency respectively after a 5 min UV exposure (Figure 3). This high efficiency is primarily attributed to the inclusion of two photocleavable linkers positioned between the nanobody and streptavidin, each designed for over 90% cleavage efficiency. During the nine-cycle reiterative staining, we further quenched residual fluorescence and autofluorescence on the tissue sections by using a H_2_O_2_-based solution (25 mL PBS + 4.5 mL 30% H_2_O_2_ + 0.8 mL 1 M NaOH for 30 min) to facilitate the detection of low-abundance proteins. We did not observe cross-cycle contamination signals which would have shown different distributions of many kidney structure proteins. Our approach using UV exposure and a mild quenching solution avoids the use of harsh chemicals on the tissue; consequently, no obvious tissue damage or decrease in fluorescence intensity was observed during the cyclic experiment. Using a slide scanner for whole tissue quantification, the intensities of proteins such as megalin and alpha SMA in the 7th and 9th cycles remained comparable to those observed in samples without reiterative staining. Importantly, reiterative staining with MIST Linker reduces processing time by 50% for multiplex techniques that typically require approximately one week to analyze over 20 markers. In addition, the MIST Linker method requires no signal amplification and is compatible with antibodies from a wide variety of sources.

**Figure 4:**
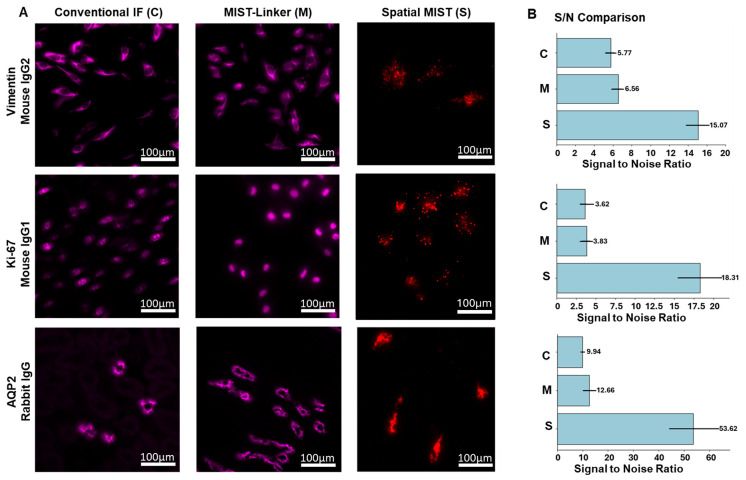
Nanobody-based Spatial MIST enables versatile staining across multiple antibody isotypes. (**A**) Representative fluorescence images generated by conventional 2-step IF method (C), MIST Linker IF method (M), and spatial MIST detection (S) for vimentin, Ki-67, and aquaporin-2 (AQP2). (**B**) Bar plots of signal-to-noise ratios for those methods and proteins. Error bars represent the standard deviation (SD) of means.

Detection of these biomarkers by spatial MIST with the MIST Linker method achieves substantially higher signal-to-noise (S/N) ratios. Consistent with the results shown in Figure 2, proteins such as Vimentin, Ki-67, and AQP2 exhibit slightly higher S/N ratios with MIST Linker labeling compared to conventional immunofluorescence (IF), while maintaining highly concordant spatial features (Figure 4). The S/N values for conventional IF span approximately 3–12, which is typical for standard laboratory settings, whereas spatial MIST markedly enhances S/N to values exceeding 15, reaching as high as 53.62 for AQP2. This improvement is primarily attributed to the negligible autofluorescence of the MIST array. In Figure 4, the MIST arrays incorporate two types of DNA-encoded microbeads, each designed to simultaneously detect two distinct DNA oligonucleotides, enabling the specific capture of two UV-released oligonucleotides with different fluorescent labels. The spatial MIST workflow includes an additional ~10 min signal transfer step, during which UV-cleaved DNA oligonucleotides are locally transferred and hybridized to complementary sequences on the MIST array (Figure 1E). The array can then be directly imaged without further signal amplification, in contrast to our established spatial MIST approach, which relies on chemical antibody–DNA conjugation and requires an additional amplification step and specialized DNA designs for dual-color detection, adding approximately 2 h to the protocol [24]. In all, with the signal intensities comparable to those obtained by conventional IF and current spatial MIST, MIST Linker enables significant saving of testing time, which is particularly critical to high-multiplex assays.

### 3.2. MIST Linker in Combination with Spatial MIST for Spatial Proteomics

**Figure 5:**
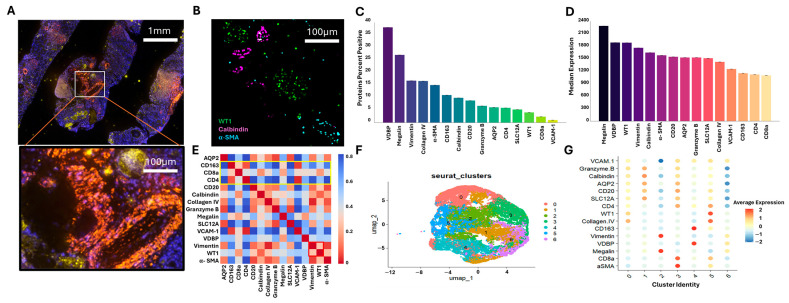
Multiplexed spatial MIST analysis of a human kidney biopsy sample. A total of 15 proteins were measured and used for single-cell expression and spatial analysis. (**A**) Raw image on MIST array after spatial MIST assay. (**B**) Reconstructed images of representative proteins by spatial MIST multiplex detection. (**C**) Bar plot of ranked marker-positive cells by the percentage for each protein. (**D**) Bar plot of ranked median expression intensity in positive cells for each protein. (**E**) Heatmap of average normalized protein spatial distances across the 15 measured proteins. (**F**) UMAP of Seurat-based single-cell clustering colored by cluster identity. (**G**) Dot plot showing protein expression across Seurat-defined clusters. Color indicates relative expression level, with red representing higher expression and blue representing lower expression.

Spatial MIST is built upon two core components: antibody–oligonucleotide conjugates for immunostaining and MIST array for localized detection. We have thoroughly optimized the MIST array in our previous work [24,25]. MIST Linker has been evaluated as the surrogate of our legacy antibody–oligonucleotide conjugates in spatial proteomic studies on various sample sources. For multiplex assays, we saturate the antibodies with sufficient MIST Linker to prevent potential crosstalk when applying a cocktail of conjugates to a tissue sample. The excess MIST Linker and DNA have been removed by the purification process. We first applied the platform to a human kidney biopsy sample from a patient with diabetic kidney disease (DKD). In the raw MIST array image (Figure 5A), the overall tissue architecture is clearly preserved and readily visualized, indicating that the assay captures spatial information with high resolution directly on the array. In the reconstructed spatial MIST image (Figure 5B), WT1 (green) distinctly localizes to the glomerulus, Calbindin (magenta) highlights distal convoluted tubules, and aSMA (cyan) marks vessel-like structures consistent with blood vessels, demonstrating that spatial MIST can resolve individual renal microarchitectures with clear protein-specific spatial fidelity.

The bar plots in Figure 5C,D further show that spatial MIST provides quantitative information on both the proportion of marker-positive cells and the expression intensity of the 15 measured proteins respectively in the kidney cortical region. VDBP (37.0% positive) and megalin (25.5% positive), both tubular markers, present the highest percentages of marker-positive cells. Within the marker-expressing cells, Megalin, VDBP, and WT1 exhibit the highest median expression levels, with median intensities of 2300, 1800, and 1780, respectively. WT1 expression was restricted to glomerular regions (4% positive; Figure 5B), which is consistent with its lower overall representation across the full tissue section [31,32]. However, the strong WT1 signal suggests that glomerular structure and integrity were also preserved [31,32,33]. To identify spatial relationships between protein pairs, a spatial proximity heatmap was generated (Figure 5E) by determining the centroid of each cell expressing a protein and calculating the Euclidean distance between them within the kidney cortical region. From this panel of 15 proteins, a total of 105 protein pairwise distances were calculated with a median normalized spatial distance of approximately 0.4. Distances above 0.4 were considered spatially far (colored in blue) while distances below this were considered close. The immune markers CD4, CD8a, and CD163 (highlighted in yellow) were spatially distant from most other proteins, with mean normalized spatial distances of 0.61, 0.45, and 0.59, respectively. In contrast, WT1 and Vimentin (highlighted in red) were found in closer spatial proximity with a spatial distance of 0.21, consistent with the expected biology of podocytes where WT1 and Vimentin are co-expressed [34,35]. Finally, in the UMAP analysis (Figure 5F) a total of seven clusters were identified from processing approximately 29,000 cells. The UMAP revealed distinct single-cell clusters while the accompanying dot plot (Figure 5F) shows the markers each cluster corresponds to.

**Figure 6:**
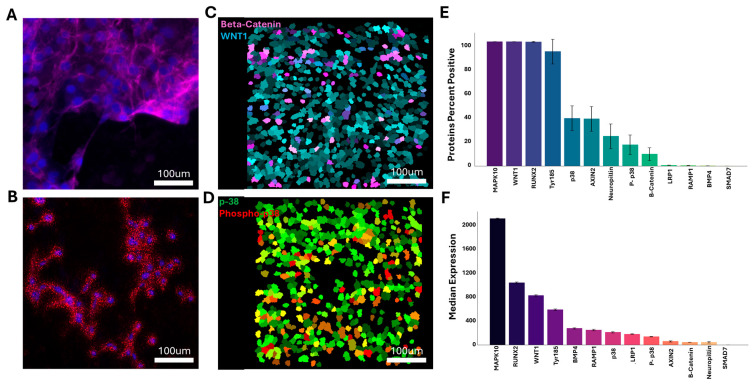
Multiplexed spatial MIST analysis of the MC3T3 cell line. A total of 13 proteins were measured and used for single-cell expression and spatial analysis. (**A**) Immunostaining images of β-catenin (magenta) and POPO-1 (blue; nuclear staining). (**B**) Raw MIST array image of β-catenin and POPO-1 distribution by spatial MIST assay. (**C**,**D**) Reconstructed images of representative proteins in single cells by spatial MIST multiplex detection. β-catenin in magenta and WNT1 in cyan are shown in (**C**); p38 in green and phospho-p38 in red are shown in (**D**). (**E**) Bar plot of ranked marker-positive cells by the percentage for each protein. (**F**) Bar plot of ranked median expression intensity in positive cells for each protein. Error bars indicate the standard deviation of two repeats of spatial MIST assays.

Last, we chose a cell line to further demonstrate the applicability of spatial MIST beyond tissue by applying the platform to the MC3T3 cell line using a 13-protein panel. The immunostaining image in Figure 6A shows the expected cellular localization of β-catenin together with nuclear staining by POPO-1, providing a reference for comparison with the spatial MIST readout. Following clamping, the corresponding raw microarray image (Figure 6B) preserved the overall cellular distribution of these signals, indicating that the array-based transfer step retained spatial information at the cellular level. The spatial MIST reconstructed images in Figure 6C,D further demonstrate that the platform can resolve distinct protein localization patterns in cultured cells. In Figure 6C, β-catenin (magenta) and WNT1 (cyan) showed broad but heterogeneous spatial distributions across the MC3T3 cell population, consistent with their involvement in Wnt-associated signaling [36,37]. In Figure 6D, p38 (green) and phospho-p38 (red) exhibited overlapping yet distinct patterns, reflecting the ability of spatial MIST to distinguish both total protein abundance and phosphorylation-associated signaling states within the same sample [38].

The quantitative plots in Figure 6E,F demonstrate robust single-cell protein profiling across the 13-marker panel for two samples of the same cell line. MAPK10, WNT1, and RUNX2 were positive in nearly all cells, while Tyr185 was also broadly expressed, with approximately 94% marker-positive cells across the MC3T3 population. MAPK10 also showed the highest median expression (2080), followed by RUNX2 (1020), WNT1 (820), and Tyr185 (580), suggesting that these markers were both highly prevalent and strongly expressed in this cell line. Importantly, these results were obtained from two separate cultures, and the relatively low standard deviations (maximum SD ± 10; average SD ± 8) across the single-cell proteins support good consistency and reproducibility between spatial MIST assays. Overall, these data further demonstrate the broad applicability of spatial MIST across distinct sample types, enabling integrated analysis of protein localization, multiplex expression profiling, and single-cell heterogeneity in both tissue specimens and cell lines.

## 4. Conclusions

In this study, we introduce the MIST Linker, a rapid and site-specific antibody conjugation strategy that enables efficient attachment of fluorophores and DNA oligonucleotides to the Fc region of antibodies using minimal input. This approach preserves antigen-binding activity while improving staining performance, yielding results comparable to or exceeding those of conventional immunohistochemistry (IHC). It also overcomes key limitations of conventional conjugation methods, including stochastic labeling and high antibody input requirements. When integrated with the spatial MIST platform, the MIST Linker enables sensitive, high-resolution, and highly multiplexed protein detection across diverse sample types, including human tissues and cultured cells. The incorporation of UV-cleavable linkers further supports reiterative staining, enabling efficient cyclic imaging without compromising tissue integrity. Together, this platform provides a scalable and versatile solution for spatial proteomics with broad applications in biological research, biomarker discovery, and clinical diagnostics. Future work will further evaluate MIST Linker scalability, including batch-to-batch consistency, reagent recovery, storage stability, and freeze–thaw tolerance to support larger-scale spatial proteomic studies.

## Data Availability

Data available upon request.

## Supplementary Materials

The following supporting information can be downloaded at https://www.mdpi.com/article/10.3390/bios16070385/s1: Figure S1: 20x zoomed brightfield image of Spatial MIST microarray. Beads depicted are 2 um polystyrene microbeads which are uniformly distributed into a monolayer. Figure S2: Native gel electrophoresis analysis of MIST Linker assembly. (A) Native gel analysis of blank, antibody only, and antibody with MIST Linker. The blank lane showed no visible band, while the antibody only lane showed a band at approximately 165 kDa. The antibody and MIST Linker complex showed a shifted band at an apparent molecular weight of approximately 305 kDa. (B) Time course analysis of MIST Linker assembly from 30 s to 20 min. The assembled complex band was observed at approximately 305 kDa at all tested time points, with no additional observable unassembled antibody or intermediate bands, indicating rapid assembly within 30 s under these conditions. Figure S3: Negative control staining with Rabbit IgG MIST Linker on mouse tissue. Mouse tissue was stained with Rabbit IgG MIST Linker only, without primary antibody. No detectable fluorescence signal was observed, indicating minimal nonspecific binding of the linker under these staining conditions. Figure S4: Comparison of Nestin staining in mouse kidney glomeruli using conventional IHC and MIST Linker labeling. Representative images of mouse tissue show that Nestin staining morphology and glomerular localization were preserved with MIST Linker labeling compared with conventional IHC, despite reduced raw fluorescence intensity. Quantification of representative raw intensity showed 1180 AU for conventional IHC and 550 AU for MIST Linker staining. Scale bars: 100 µm. Table S1: (a) Oligo Sequences Conjugated to Microbeads; (b) Oligo Sequences for MIST Linker to tag antibodies. Table S2: Antibody Panels. (a) Kidney Panel: 15 Proteins; (b) MC3T3 Cell Line Panel: 13 Proteins. Table S3: Decoding color scheme used to identify each protein. (a) Kidney Panel Decoding; (b) MC3T3 Cell Line Panel Decoding.
