# Supplementary material for: Universal, Rapid, and Cleavable Labeling of Antibodies by Fluorophores and DNA Oligonucleotides for Multiplex Immunostaining and Spatial Proteomics Through MIST Linker
Source: Biosensors (Basel). 2026 Jul 15;16(7):385. doi: 10.3390/bios16070385 (PMC13407159; doi:10.3390/bios16070385)
Supplement: Supplementary file 1 [file biosensors-16-00385-s001.zip › biosensors-4369100-supplementary.pdf]

Supplementary Information for

Universal, Rapid and Cleavable Labeling of Antibodies by Fluorophores and DNA Oligonucleotides for Multiplex Immunostaining and Spatial Proteomics through MIST Linker

Arafat Meah[1], Shuo Yin[1], Saimoen Anderson[1], Ming Lin[2], Shuo Liang[1], Meghana Davuluri[3], Yi-Xian Qin[3], Sandeep K. Mallipattu[4,5], Jun Wang[1,6*]

[1]Multiplex Biotechnology Laboratory, Department of Biomedical Engineering, State University of New York at Stony Brook, Stony Brook, NY 11794, USA

[2]Department of Computer Science, State University of New York at Stony Brook, Stony Brook, NY 11794, USA.

[3]Department of Biomedical Engineering, State University of New York at Stony Brook, Stony Brook, NY 11794, USA

[4]Division of Nephrology and Hypertension, Department of Medicine, Stony Brook School of Medicine, Stony Brook, NY 11794, USA

[5]Renal Section, Northport VA Medical Center, Northport, NY 11768, USA

[6]MIST Bioscience, New York, USA

[#]Corresponding Authors

Email: Jun.wang.5@stonybrook.edu

1. **Supplementary Figure**

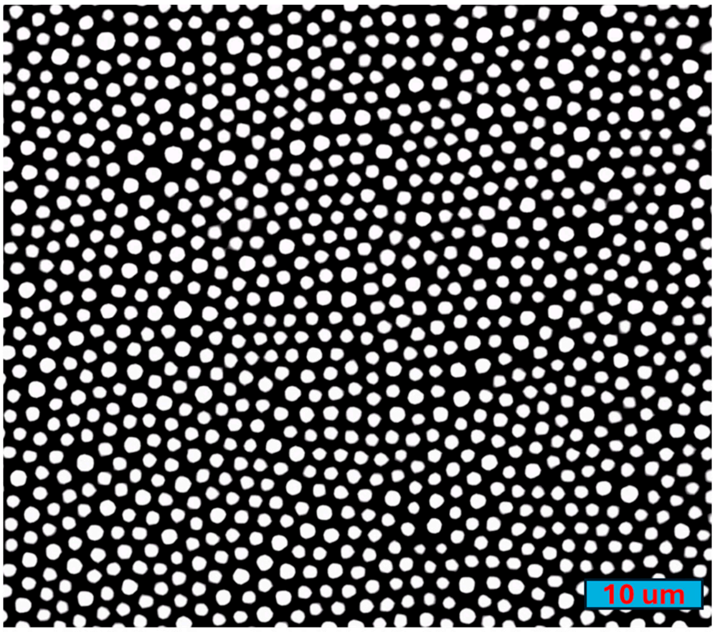

Figure S1: 20x zoomed brightfield image of Spatial MIST microarray. Beads depicted are 2 um polystyrene microbeads which are uniformly distributed into a monolayer.

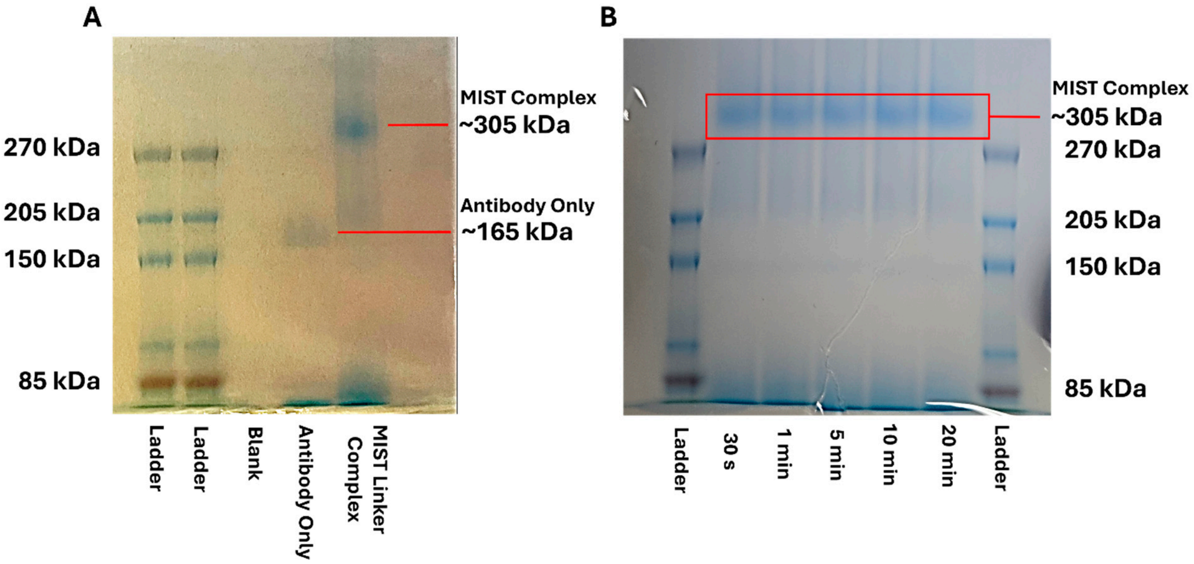

Figure S2: Native gel electrophoresis analysis of MIST Linker assembly. (A) Native gel analysis of blank, antibody only, and antibody with MIST Linker. The blank lane showed no visible band, while the antibody only lane showed a band at approximately 165 kDa. The antibody and MIST Linker complex showed a shifted band at an apparent molecular weight of approximately 305 kDa. (B) Time course analysis of MIST Linker assembly from 30 s to 20 min. The assembled complex band was observed at approximately 305 kDa at all tested time points, with no additional observable unassembled antibody or intermediate bands, indicating rapid assembly within 30 s under these conditions.

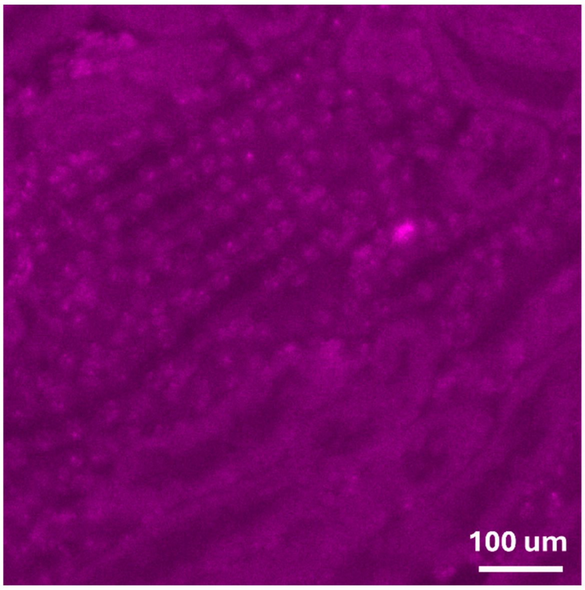

Figure S3: Negative control staining with Rabbit IgG MIST Linker on mouse tissue. Mouse tissue was stained with Rabbit IgG MIST Linker only, without primary antibody. No detectable fluorescence signal was observed, indicating minimal nonspecific binding of the linker under these staining conditions.

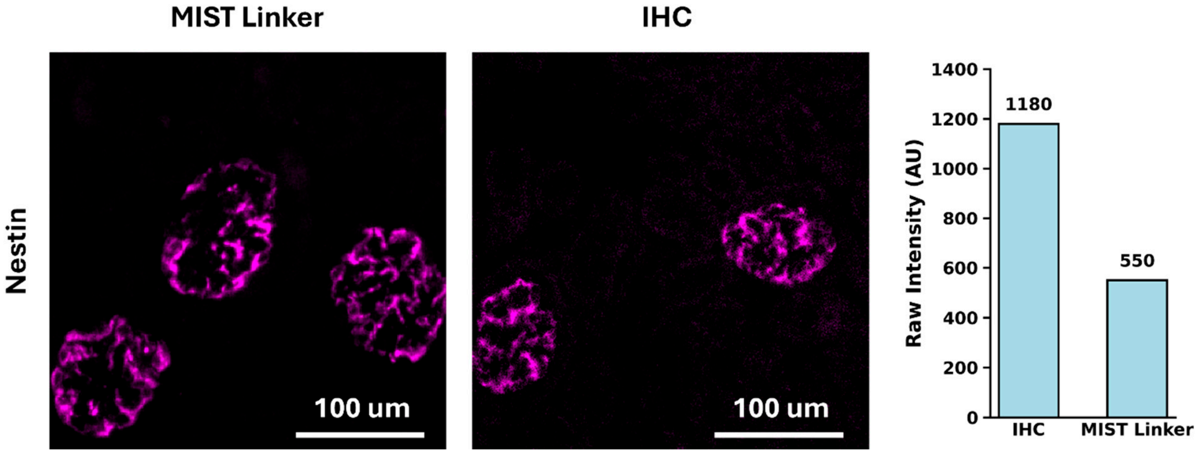

Figure S4: Comparison of Nestin staining in mouse kidney glomeruli using conventional IHC and MIST Linker labeling. Representative images of mouse tissue show that Nestin staining morphology and glomerular localization were preserved with MIST Linker labeling compared with conventional IHC, despite reduced raw fluorescence intensity. Quantification of representative raw intensity showed 1180 AU for conventional IHC and 550 AU for MIST Linker staining. Scale bars: 100 μm.

2. Table Supplement

Table S1: (a) Oligo Sequences Conjugated to Microbeads; (b) Oligo Sequences for MIST Linker to tag antibodies

**(a)**	
**Sequence Name**	**Sequence (5-->3)**
Seq1	/5AmMC6/ATG TCT TCC GGC GCA
Seq2	/5AmMC6/AAT GAG TGC GCG GTC
Seq3	/5AmMC6/GGA TTG ACG CAG GCA
Seq4	/5AmMC6/CAA GAG CGC CGA ACT
Seq5	/5AmMC6/AGT TGG TGC CTG GTG
Seq6	/5AmMC6/CCG GCG AGT TCT GTT
Seq7	/5AmMC6/TCC GGC CAC TCT ACT
Seq8	/5AmMC6/ATG GTT GGA CGG AGC
Seq9	/5AmMC6/TTC GGC ACA CAG AGG
Seq10	/5AmMC6/TTC ACT CGT CGG CTG
Seq11	/5AmMC6/ACG ACG AAG CCA CTC
Seq12	/5AmMC6/GCT CTC GAC GTG TTC
Seq13	/5AmMC6/CGG AAC CAG GAA GCT
Seq14	/5AmMC6/AGA GTA CGC GCT TCC
Seq15	/5AmMC6/CCA GGT CAG CGA GTT
Seq16	/5AmMC6/GCA AGC ACT CGT TCG
Seq17	/5AmMC6/GCA GGC TAG GTC GAA
Seq18	/5AmMC6/ATC CAA GCG CGA GAA
Seq19	/5AmMC6/ACG CTC CTC CTC ATG
Seq20	/5AmMC6/AGT CTC TCG GCC ATG
Seq21	/5AmMC6/TTC AGT TCA CGG CCA
Seq22	/5AmMC6/CGC CGG TTG AGA TTG
Seq23	/5AmMC6/ATC ACT GCC GAC GAA
Seq24	/5AmMC6/AGC CAC TTC ACA CCA
Seq25	/5AmMC6/GAC CTC GTA CCA CCT
(b)	
**Sequence Name**	**Sequence (5-->3)**
Seq1'	/5Biosg/TGC GCC GGA AGA CAT /3AmMO/
Seq2'	/5Biosg/GAC CGC GCA CTC ATT /3AmMO/
Seq3'	/5Biosg/TGC CTG CGT CAA TCC /3AmMO/
Seq4'	/5Biosg/AGT TCG GCG CTC TTG /3AmMO/
Seq5'	/5Biosg/CAC CAG GCA CCA ACT /3AmMO/

Seq6'	/5Biosg/AAC AGA ACT CGC CGG /3AmMO/
Seq7'	/5Biosg/AGT AGA GTG GCC GGA /3AmMO/
Seq8'	/5Biosg/GCT CCG TCC AAC CAT /3AmMO/
Seq9'	/5Biosg/CCT CTG TGT GCC GAA /3AmMO/
Seq10'	/5Biosg/CAG CCG ACG AGT GAA /3AmMO/
Seq11'	/5Biosg/GAG TGG CTT CGT CGT /3AmMO/
Seq12'	/5Biosg/GAA CAC GTC GAG AGC /3AmMO/
Seq13'	/5Biosg/AGC TTC CTG GTT CCG /3AmMO/
Seq14' - **K**	/5/GGA AGC GCG TAC TCT TAA TCT **AAT TCT GGT CGC GG /3AmMO/**
Seq15' - **K**	/5/AAC TCG CTG ACC TGG TAA TCT **AAT TCT GGT CGC GG /3AmMO/**
Seq16' - **K**	/5/CGA ACG AGT GCT TGC TAA TCT **AAT TCT GGT CGC GG /3AmMO/**
Seq17' - **K**	/5/TTC GAC CTA GCC TGC TAA TCT **AAT TCT GGT CGC GG /3AmMO/**
Seq18' - **K**	/5/TTC TCG CGC TTG GAT TAA TCT **AAT TCT GGT CGC GG /3AmMO/**
Seq19' - **K**	/5/CAT GAG GAG GAG CGT TAA TCT **AAT TCT GGT CGC GG /3AmMO/**
Seq20' - **K**	/5/CAT GGC CGA GAG ACT TAA TCT **AAT TCT GGT CGC GG /3AmMO/**
Seq21' - **K**	/5/TGG CCG TGA ACT GAA TAA TCT **AAT TCT GGT CGC GG /3AmMO/**
Seq22' - **K**	/5/CAA TCT CAA CCG GCG TAA TCT **AAT TCT GGT CGC GG /3AmMO/**
Seq23' - **K**	/5/TTC GTC GGC AGT GAT TAA TCT **AAT TCT GGT CGC GG /3AmMO/**
Seq24' - **K**	/5/TGG TGT GAA GTG GCT TAA TCT **AAT TCT GGT CGC GG /3AmMO/**
Seq25' - **K**	/5/AGG TGG TAC GAG GTC TAA TCT **AAT TCT GGT CGC GG /3AmMO/**

Table S2: Antibody Panels. (a) Kidney Panel: 15 Proteins; (b) MC3T3 Cell Line Panel: 13 Proteins.

(a)		
**Antigen**	**Host Species**	**Cat No**

aSMA	Mouse IgG2	14-9760-82
Calbindin	Rabbit/IgG	PA5-85669
Vimentin	Mouse IgG2	MA5-11883
Megalin	Mouse IgG1	MABS489
WT1	Rabbit/IgG	PA5-116131
AQP2	Rabbit/IgG	PA5-38004
CD20	Mouse IgG2a	302302
VDBP	Rabbit / IgG	16922-1-AP
Collagen IV	Mouse IgG2b, k	14-9871-82
Granzyme B	Mouse IgG2A	MAB2906
CD4	Mouse IgG1, k	300502
CD8a	Mouse IgG1, κ	372902
CD163	Mouse IgG1, κ	364302
VCAM-1	Mouse IgG1, k	919801
SLC12A	Rabbit/IgG	PA5-80002

(b)

**Antibody name**	**Host / isotype**
WNT1	Rabbit IgG (polyclonal)
RAMP1	Rabbit IgG (polyclonal)
LRP1	Rabbit IgG (polyclonal)
MAPK10	Rabbit IgG (polyclonal)
SMAD7	Rabbit IgG (polyclonal)
Phospho-p38 MAPK (Thr180/Tyr182)	Rabbit IgG (polyclonal)
p38 MAPK	Rabbit IgG (polyclonal)
RUNX2	Rabbit IgG (recombinant)
Phospho-JNK (Tyr185)	Rabbit IgG (recombinant)
Neuropilin 2	Rabbit IgG (polyclonal)
Beta Catenin	Rabbit IgG (polyclonal)
BMP4	Rabbit IgG (polyclonal)
AXIN2	Rabbit IgG (polyclonal)

Table S3: Decoding color scheme used to identify each protein. (a) Kidney Panel Decoding; (b) MC3T3 Cell Line Panel Decoding

(a)		
Protein name and Sequence	Code (0: GFP channel; 1: Cy3 channel; 2: Cy5 channel. 3: Cy7 channel; From cycle 1, cycle 2 to cycle3)	
Seq 1' a-SMA	3	3
Seq 2' Calbindin	3	2
Seq 3' SLC12A	0	0
Seq 4' WT1/Seq 11' CD4	0	1
Seq 5' Vimentin/Seq 12' CD8a	1	3
Seq 6' Megalin/Seq 13' AQP2	1	0
Seq 7' CD20/Seq 14' VDBP	1	1
Seq 8' VCAM-1/Seq 15' CD163	2	2
Seq 9' Collagen IV	2	3
Seq 10' Granzyme B	2	0

(b)

Protein name and Sequence	Code (0: GFP channel; 1: Cy3 channel; 2: Cy5 channel. 3: Cy7 channel;	
	**Cycle 1**	**Cycle 2**
Seq 1'/ Seq 13' WNT1/BMP4	3	3
Seq 2'/ Seq 14' RAMP1/Neuropillin	3	2
Seq 3'/ Seq 15' LRP1/AXIN2	0	0
Seq 4' MAPK10	0	1
Seq 5' SMAD7	1	3
Seq 6' Phospo-p38	1	0
Seq 7' p38	1	1
Seq 8'/ Seq 16' RUNX2/Tyr 185	2	2
Seq 9' B-Catenin	2	3
